# Cichlid Evolution: Lessons in Diversification

**DOI:** 10.4061/2011/847969

**Published:** 2011-12-05

**Authors:** Stephan Koblmüller, R. Craig Albertson, Martin J. Genner, Kristina M. Sefc, Tetsumi Takahashi

**Affiliations:** ^1^Department of Zoology, Karl-Franzens University, Universitätsplatz 2, 8010 Graz, Austria; ^2^Department of Biology, University of Massachusetts, Amherst, MA 01002, USA; ^3^School of Biological Sciences, University of Bristol, Woodland Road, Bristol BS8 1UG, UK; ^4^Graduate School of Science, Kyoto University, Kitashirakawa-Oiwake, Sakyo, Kyoto 606-8502, Japan

## 

With an estimated 3000 species, distributed from Central and South America, across Africa to Madagascar, the Middle East, and southern India, cichlid fishes (Cichlidae) represent the most species-rich family of vertebrates. In total they account for about 10% of extant teleost diversity. Throughout their distributional range, cichlids have repeatedly demonstrated their capacity for undergoing adaptive radiation, generating an outstanding variation of body shapes, color patterns, and behavior, and an enormous diversity of trophic and ecological specializations. This has made them an important model system for the field of evolutionary biology. With the completion of the first cichlid genome sequences, cichlid fishes are likely to receive even more attention in evolutionary research.

This special issue presents a collection of papers that tackle various aspects of cichlid biology essential for gaining a deeper understanding of factors and processes responsible for generating their diversity. Naturally, the selected topics and papers do not fully represent all fields of research that have contributed to our understanding of cichlid evolution. Nevertheless, they do represent rich and diverse knowledge which we are pleased to share with the readers of IJEB. Papers are summarized below in the order in which they appear in this special issue.

Lake Tanganyika is the oldest of the three East African Great Lakes and harbors the morphologically, behaviorally, ecologically, and genetically most diverse lacustrine cichlid species assemblage. In their review, “*The adaptive radiation of cichlid fish in Lake Tanganyika: a morphological perspective*,” T. Takahashi and S. Koblmüller give a brief overview about recent phylogenetic findings and provide a synthesis of morphological studies that relate to the adaptive radiation of Lake Tanganyika's cichlids. They discuss many of the relevant studies that have contributed to our increasing knowledge of the evolutionary pathways and mechanisms generating this exceptional diversity within a short period of time. Furthermore, the authors highlight the potential importance of ontogenetic changes in morphology for establishing taxa in complex species-rich communities, a largely neglected issue in East Africa's species-rich lacustrine cichlid species assemblages.

Recent advances in morphometric methods provide great potential for detailed analyses of cichlid anatomy in an evolutionary context. In particular the establishment of geometric morphometric approaches has revolutionized the analysis of morphological data in cichlid evolutionary research. In their review, “*The utility of geometric morphometrics to elucidate pathways of cichlid fish evolution*,” M. Kerschbaumer and C. Sturmbauer introduce geometric morphometric concepts and methods and also provide a summary of studies that employed this approach for analyzing and comparing cichlid morphologies. The authors promote the tremendous potential of this method for answering a variety of evolutionary questions involving complex shape changes in cichlid fish.

In the 1970s, Karel Liem demonstrated a functional decoupling between the oral and pharyngeal jaws in African cichlids. He postulated that this attribute had freed the oral jaws from the functional constraint of food processing, thereby allowing the oral jaws to evolve elaborate modifications for prey capture. Implicit to this assertion is a degree of independence of the oral jaws from the rest of the craniofacial skeleton. In their paper, “*Modularity of the oral jaws is linked to repeated changes in the craniofacial shape of African cichlids*,” K. J. Parsons et al. tested the hypothesis that the oral jaw apparatus among East African cichlids represents a variational module. Their data offer strong support for the presence of such a module across all lakes as well as within each lake. This finding helps to explain why the direction of cichlid craniofacial radiations has been largely conserved across lineages.

The cichlid species of Lake Tanganyika are also the focus of another review, “*Mating and parental care in Lake Tanganyika's cichlids*,” by K. M. Sefc. The paper starts with a comprehensive evaluation of field and genetic data on the mating and parenting behavior of mouthbrooding and substrate breeding cichlid species. The review then considers alternative reproductive tactics, including cooperative breeding, sneaking, and piracy. In the electronic format, this summary can easily be searched for information on particular species and genera. The review also addresses intraspecific variability in mating behavior, the evolution of cichlid mating systems and sexual dimorphism, and closes by emphasizing the difficulty of defining mating systems and sexual selection intensity at species level. 

It is often assumed that the primary selective force driving divergence in sexually selected traits is direct female choice. However, there is another possibility: indirect selection operating through male-male competition. In “*Male-male competition as a force in evolutionary diversification: evidence in haplochromine cichlid fish*,” P. D. Dijkstra and T. Groothuis review the role of male-male competition in cichlid divergence, focusing upon the haplochromine cichlids of Africa. They use examples to show how males with novel color patterns in a community may enjoy an advantage as they are subject to less aggression from established territorial males and speculate that this may contribute to speciation by allowing males with novel colors to establish territorial space in communities. They also discuss how aggression biases are not always symmetric between sympatric color morphs, and describe the evidence that the color of individuals and their aggression biases are genetically linked. The paper closes with intriguing proposals for future work. These include tests to establish how male-male competition and aggression biases are mediated by the availability of territorial space, and how selection on traits correlated with color, for example, physiological traits, may promote or constrain adaptive evolution.

The influence of male color pattern on female mate choice was examined in a research paper by O. Svensson, B. Egger and others, “*Segregation of species-specific male attractiveness in F2 hybrid Lake Malawi cichlid fish.*” In laboratory experiments with two sympatric, differently colored *Pseudotropheus* species, females were given a choice of hybrid males displaying a range of intermediate color patterns, and male mating success was determined by genetic analysis of broods. Although males differed in their attractiveness towards females, there was no correlation between male mating success and male coloration, and mate choice may have depended on nonvisual signals, segregating male preferences, or visual signals not quantified in the study. A quantitative genetic model estimated that only as few as two chromosomal regions may control species-specific attractiveness, which suggests that few genes with major effects can promote reproductive isolation in the radiation of Lake Malawi cichlids. 

Environmental influences on color pattern evolution are the focus of a study on another Lake Malawi cichlid genus. In “*One fish, two fish, red fish, blue fish: geography, ecology, sympatry, and male coloration in the Lake Malawi cichlid genus Labeotropheus (Perciformes: Cichlidae),*” M. J. Pauers identified biases in the distribution of red coloration dependent on lake region, depth distribution, and the presence of a congeneric species. The latter observation connects to the two previous papers, as in areas of sympatry, color pattern differentiation may follow from intrasexual competition between similarly colored males or the requirement of mate recognition cues to maintain reproductive isolation. The correlations detected in this study underscore the importance of both sexual and natural selection for nuptial color pattern evolution. An interesting postscript to the discussion deals with the question of why fish employ red coloration rather than, for example, becoming ever bluer in their endeavor to signal efficiently to conspecifics lacking long-wavelength-sensitive photoreceptors, in a long-wavelength-deprived environment. M. J. Pauers suggests that the answer lies in within-pattern contrast and supports his claim with an instructive illustration of a protanopic's (human or fish) impression of *Labeotropheus* coloration. 

The quantitative genetics underlying “animal personality” is expressed in the behavioral variation within and between individual animals and has hitherto been examined mainly in domesticated species. In “*Repeatability and heritability of behavioral types in a social cichlid*,” N. Chervet and coauthors used the cooperatively breeding cichlid *Neolamprologus pulcher* to study long-term repeatability and heritability of behavioral types in a wild animal. Boldness, aggressiveness, and exploration propensity were repeatedly scored in 1779 individuals up to 1201 days apart and joined into a single measure of behavioral type. Despite considerable repeatability (which declined over time), low heritability suggested other, perhaps nongenetic, effects on the variability of behavioral type. Importantly, the paper identifies several methodological and conceptual issues relevant for studies on correlated behaviors, for instance, the need to determine repeatability and to assess the fitness effect of the studied behavior in a natural situation. 

A familiar concept among evolutionary biologists studying cichlids is that philopatry of fish populations provides opportunities for local selective forces to drive evolution in allopatry. There is now a considerable amount of evidence for this among rock-dwelling lacustrine cichlids, but less so for species that occupy offshore habitats, or peripheral lagoons. In “*Low genetic and morphometric intraspecific divergence in peripheral Copadichromis populations (Perciformes: Cichlidae) in the Lake Malawi basin*,” D. Anseeuw and coauthors tested for morphological and genetic divergence among populations of *Copadichromis* species in the Lake Malawi basin. They found that populations of both species showed slight genetic differences, but there was no clear evidence that the population in the peripheral Lake Malombe differed substantially in neutral genetic markers or body shape to the populations in the main lake. Perhaps one of the most striking aspects of the study is that the body sizes of both species sampled from Lake Malombe were considerably smaller than those in Lake Malawi. Given that Lake Malombe has been heavily fished for decades, the authors discuss the intriguing possibility that the observed contrast in body size is an evolutionary response to fisheries exploitation.

Recent evidence suggests that diversification and maintenance of divergence despite gene flow are much more common than previously assumed. In “*Community genetics reveal elevated levels of sympatric gene flow among morphologically similar but not among morphologically dissimilar species of Lake Victoria cichlid fish*,” N. Konijnendijk et al. demonstrate this phenomenon by examining the genetic structure among populations of five species of Lake Victoria cichlids in each of four island communities. They show that allopatric conspecific populations of these five species are on average more strongly differentiated than sympatrically occurring heterospecific populations of morphologically similar species, consistent with the idea that maintenance or maybe even evolution of phenotypic divergence is possible in sympatry despite high levels of interspecific gene flow. Hence, large parts of the genome might be exchanged among species without dramatically affecting morphology. On the other hand, phenotypic similarity between conspecific populations from different islands can be maintained in the face of very little gene flow between these conspecific populations indicating that geographic isolation is apparently not the sole most important determinant of diversification in recently diverged (Lake Victoria) cichlids.

The classification of Discus (genus *Symphysodon*) populations into species has been a difficult and somewhat controversial subject. In their study “*A molecular perspective on systematics, taxonomy and classification of Amazonian Discus fishes of the genus Symphysodon*,” M. V. Amado et al. investigated this issue using wide ranging sampling of 24 populations across the Amazon basin, comparing their genetic structure using a suite of 13 microsatellite loci. Their results showed the presence of four nuclear genetic groups, each largely corresponding to color morphs. Because of the broad scale of sampling, it was also possible to demonstrate that these genetic groups also differed in the average habitat use, a pattern consistent with evolutionary divergence in habitat preferences. Together the study clearly demonstrates the value of a broad-scale sampling and the use of multiple molecular markers to test hypotheses of species status and inform systematic revisions.

The exact age of origin of and diversification events within cichlid fishes is still a matter of discussion, and several alternative hypotheses have been put forward in recent years. In their strategy paper “*The monogenean parasite fauna of cichlids: a potential tool for host biogeography*,” A. Pariselle et al. propose an alternative approach for tackling the origin and phylogeographic history of cichlid fishes. They suggest that phylogenetic and biogeographical analyses of dactylogyrid parasites might be helpful in elucidating the history of their cichlid hosts and discuss a preliminary morphology-based phylogenetic analysis on the genus level which fails to support one specific scenario. Hence, the authors emphasize that a much more comprehensive in-depth analysis including molecular data and parasites from various host families will be essential for providing enough resolution to contribute to solving this heavily discussed issue in cichlid evolutionary research.

Altogether, this is a diverse array of papers that demonstrate the breadth of research questions that are possible to address using the cichlid system. They also show how cichlid research can significantly contribute to development and understanding of evolutionary theory. We hope you enjoy reading the contributions.

